# Refining imaging parameters for dual‐energy cone‐beam computed tomography in image‐guided radiation therapy

**DOI:** 10.1002/acm2.70516

**Published:** 2026-04-23

**Authors:** Hyejoo Kang, Andrew Keeler, Matthew Georgesen, Jason Luce, Ha Nguyen, Mathias Lehmann, Sebastien Gros, John C. Roeske

**Affiliations:** ^1^ Department of Radiation Oncology, Stritch School of Medicine, Cardinal Bernardin Cancer Center Loyola University of Chicago Maywood Illinois USA; ^2^ Department of Medical Physics Creighton University Omaha Nebraska USA; ^3^ Varian Imaging Laboratory Baden Switzerland

**Keywords:** dual energy, low imaging dose, virtual monoenergetic images

## Abstract

**Purpose:**

To develop a dose estimation method for virtual monoenergetic images (VMIs) derived from sequential dual‐energy (DE) cone‐beam computed tomography (CBCT), enhancing image quality while reducing imaging dose. The goal is to generate VMIs with lower imaging dose than standard CBCT for future clinical applications.

**Materials and Methods:**

Normalized air kerma (K_air_) was measured using an ion chamber for eight CBCT datasets with varying exposures and framerates at 80 and 140 kVp. Correlations between K_air_ and measured cone‐beam dose indices (CBDI) were established to estimate K_air_‐based imaging dose for DE‐CBCT, and then the estimates were subsequently validated. Separately, VMIs were reconstructed from eight new DE‐CBCT protocols of a Catphan 604 phantom using the Feldkamp‐Davis‐Kress (FDK) algorithm within the open‐source TIGRE. Image quality of these VMIs at 60 keV, optimal for soft tissue contrast, was evaluated using the contrast‐noise‐ratio (rCNR) relative to the clinical Pelvis Large Protocol (PLP), the mean Hounsfield units (HU) accuracy over all material inserts, and HU uniformity.

**Results:**

The average difference between estimated and measured K_air_ was 0.7 ± 2.1% for 80 kVp and 1.3 ± 1.9% for 140 kVp. All VMIs exhibited rCNR values greater than 1 (range: 1.11–1.70), indicating enhanced soft tissue contrast compared to the PLP. The estimated relative K_air_ for these VMIs ranged from 60% to 100% of a single PLP. VMIs also exhibited improved HU accuracy, reduced HU variance, and substantially improved HU uniformity including those with 60% of the PLP imaging dose.

**Conclusion:**

This pilot study demonstrates that VMIs can improve CNR and HU uniformity while reducing imaging dose by up to 40%, relative to the PLP, without compromising HU accuracy. Our approach offers potential for optimizing VMIs by balancing image quality enhancement and dose reduction. Future work will focus on the application of advanced reconstruction algorithms to further improve VMIs quality.

## 1 INTRODUCTION

Image‐guided radiation therapy (IGRT) using cone‐beam computed tomography (CBCT) has become a standard of care for radiation therapy (RT) across various disease sites, including head and neck, lung, gynecological, and prostate cancers.^1^, ^2^, ^3^, ^4^, ^5^, ^6^

There is a growing need to improve the image quality of CBCT, as it is increasingly utilized for adaptive RT (ART), where accurate soft tissue delineation is essential.^7^, ^8^, ^9^, ^10^ Furthermore, substantial improvement in soft tissue contrast of CBCT could enhance the performance of auto‐segmentation on CBCT, thereby aiding more accurate and efficient ART workflows.^11^, ^12^, ^13^

One approach to address the challenge of low soft tissue contrast is the development of dual‐energy CBCT (DE‐CBCT) protocols, which can be used to create virtual monoenergetic images (VMIs).^14^ VMIs offers the potential to enhance soft tissue visualization at specific energy levels.^15^ Ongoing technical advancements in DE‐CBCT including fast KV‐switching, dual‐layer detector, and KV filter‐based are active research domains with promising results for future clinical application.^14^, ^16^, ^17^, ^18^, ^19^, ^20^, ^21^ Despite these benefits, clinical implementation of DE‐CBCT raises concerns about increased imaging dose. Additional radiation exposure to the patient may increase radiation‐related risk, particularly with repeated imaging for patient setup.^22^, ^23^, ^24^, ^25^, ^26^, ^27^ This underscores the importance of minimizing imaging dose while maintaining clinical image quality, which is a delicate balance critical for maximizing the benefits of DE‐CBCT. Thus, developing a robust method to measure or estimate imaging dose is crucial for evaluating both image quality and imaging dose during DE‐CBCT protocol development and optimization.

Imaging dose for CT is typically quantified using standardized dose indices such as CT dose index (CTDI) and weighted CTDI (CTDIw) measured with specialized phantoms and detectors designed for diagnostic CT systems. For the purpose of CBCT imaging dose, the cone‐beam dose index (CBDI or CBDIw), analogue to CTDI and CTDIw for CT, can serve as a similar metric for dose assessment.^15^, ^28^, ^29^, ^30^, ^31^, ^32^, ^33^, ^34^ Consequently, these imaging dose measurements on CT and CBCT in RT departments are generally performed on an annual basis in accordance with AAPM recommendations.^35^ However, more frequent evaluations for CBCT may be limited by restricted access to this dedicated dosimetry equipment.

To address this limitation, a practical solution utilizing air kerma free‐in‐air (referred as “Air kerma” or “K_air_” in this paper) as a surrogate of CBDI or CBDIw has been developed for CBCT dose measurement and estimation.^36^ In contrast to the standard in‐phantom CBDI or CBDIw measurement that requires specialized equipment commonly used for diagnostic imaging dosimetry, this in air measurement method leverages detectors readily available in RT centers and enables more frequent and streamlined dose assessments during CBCT protocol optimization, and system adjustments and maintenance. Furthermore, with emerging imaging technologies such as DE‐CBCT, this approach can provide a feasible framework for dose estimation to aid in developing dose‐efficient imaging protocols with improved image quality.

Building upon the K_air_‐based dose estimation method previously established at our institution,^36^ the aim of this study is to develop clinically viable, low‐dose DE‐CBCT protocols. The objectives of this study are: (1) to establish a reliable method for estimating the imaging dose for DE‐CBCT based on K_air_ as a function of key imaging parameters such as mAs per pulse and acquisition framerate, and (2) to optimize DE‐CBCT protocols for IGRT by achieving clinically acceptable image quality while minimizing patient imaging dose.

## 2 METHODS

DE‐CBCT protocols were selected based on estimated imaging doses using linear correlations between measured CBDI/CBDIw and K_air_, ensuring the combined imaging dose remained equivalent to or lower than that of the standard clinical protocol. DE‐CBCT scans were then acquired across a range of dose levels, imaging techniques, and acquisition framerates. Subsequently, the image quality of VMIs generated from DE‐CBCT were evaluated using standard image quality metrics.

### 2.1 Imaging dose measurements for CBCT

To estimate imaging dose, reference dosimetry (CBDI and CBDIw) versus K_air_ were first plotted to assess linear correlations across CBCT protocols. This step was to evaluate the feasibility of using K_air_ as surrogate for CBDI/CBDIw. Subsequently, linear correlations were established between measured K_air_ and cumulative CBCT mAs for both 80 and 140 kVp acquisitions. These linear correlations allow for estimation of imaging dose for each DE‐CBCT protocol by referencing the K_air_ value relative to that of clinical standard CBCT protocol. This method enables dose estimation of DE‐CBCT protocols using K_air_ with varying image parameters such as the framerates and mAs per pulse.

All measurements were conducted on Varian TrueBeam linear accelerator (Varian Medical Systems, Siemens Healthineers), with an on‐board imager (OBI). Eight CBCT protocols (Table 1) were evaluated at both 80 and 140 kVp across a range of cumulative mAs and framerates. CBCT scans were acquired in half‐fan mode with a bowtie filter and a full trajectory protocol (360° rotation) for both energies. The collimator positions for kV source were x1 = −24.7 and x2 = +3.3, y1 = ‐10.7, y2 = 10.7, giving the field of view of the individual projections is 28 × 21.4 cm with 10.6 cm offset in x direction at the isocenter.

**TABLE 1:** Eight sets of CBCT protocols for image dose measurements for CBDI/CBDIw for 80 and 140 kVp. Protocol 8 for 140 kVp corresponds to Varian Pelvis Large Protocol.

Protocol	1	2	3	4	5	6	7	8
Framerate (s^−1^)	3	7	11	15	15	15	15	15
mA	75	75	75	10	45	45	65	75
Ms	25	25	25	10	10	20	20	25
Cumulative mAs	338	788	1238	90	405	810	1170	1688

**FIGURE 1:**
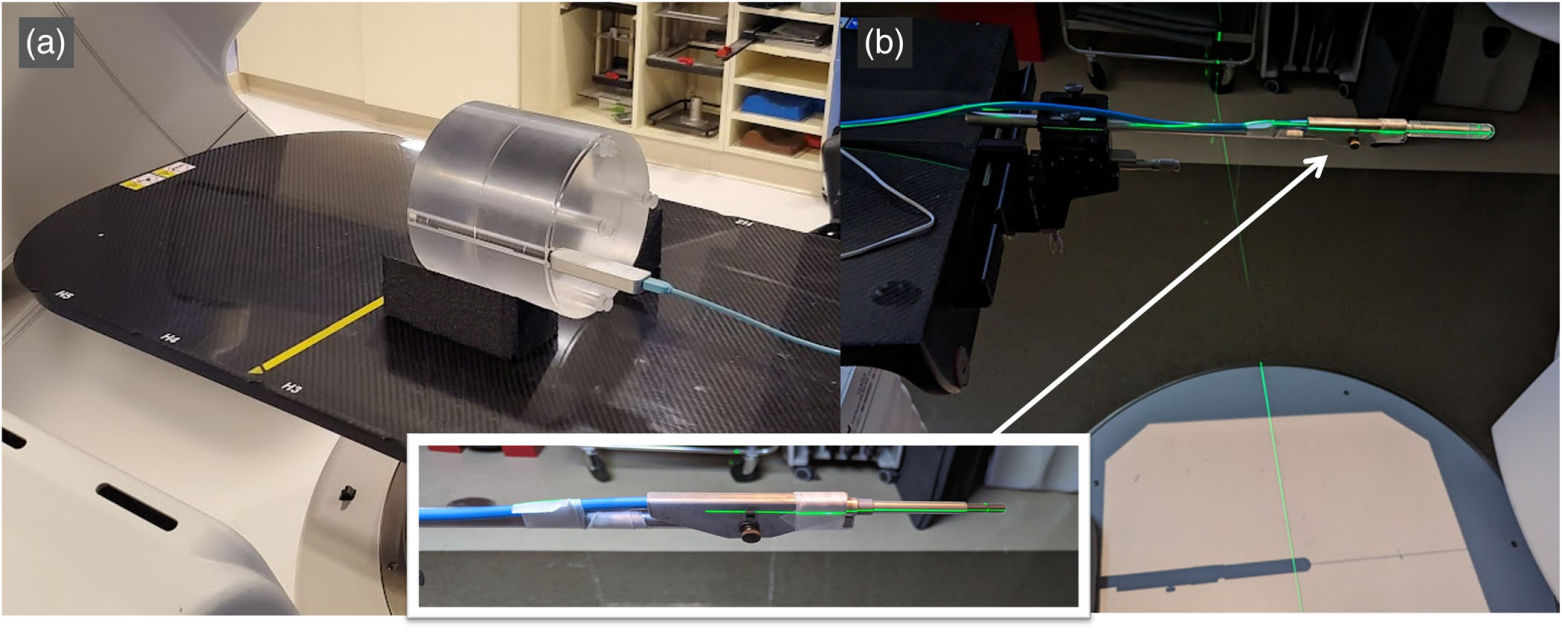
(a) Measurement setup for imaging dose, CBDIw with Raysafe X2 and PMMA cylinder head phantom. (b) Air Kerma (K_air_) measurement setup using a 0.6cc Farmer ion chamber at the isocenter aligned with room lasers and cross hair. The buildup cap was removed for the measurements as shown in the inset.

CBDI and CBDIw were measured using a 100 mm‐pencil ion chamber detector (RaySafe X2, RaySafe, USA) placed in polymethyl‐methylacrylate (PMMA) 16 cm‐diameter head cylindrical phantom for 80 kVp (Figure 1a) and a 32 cm‐diameter pelvis phantom for 140 kVp. CBDI was measured at the center of the phantom and CBDIw was measured as weighted doses at the center and four peripheral locations (one location is shown in Figure 1a). The phantom was aligned to the isocenter of the Truebeam using the room laser and the gantry crosshair.

Separately, K_air_ was measured using a farmer‐type 0.6cc ion chamber (PTW, USA) positioned at the isocenter of the TrueBeam machine (Figure 1b). The ion chamber was ADCL calibrated using an x‐ray beam at 120 kVp with the first Half‐Value Layer (HVL) of 14.73 mm Al + 1.62 mm Cu. To calculate K_air_, the calibration coefficient N_k_ (Gy/C), electrometer factor and temperature‐pressure corrections were applied to cumulative charge per CBCT protocol. Each measurement was repeated three times per protocol to ensure consistency.

To validate this K_air_‐based dose estimation, K_air_ was measured for seven DE‐CBCT protocols excluding protocol 4 in Table 1 at both 80 and 140 kVp, as the lowest setting of 90 mAs is typically not used clinically due to unacceptable image quality. For image dose comparison, all K_air_ were normalized to that of the Varian Pelvis Large Protocol (PLP).

### 2.2 Image data acquisition and reconstruction

To generate VMIs, eight new DE‐CBCT protocols of a Catphan 604 phantom (Phantomlab, NY), as listed in Table 2, were sequentially acquired at 80 and 140 kVp. For comparison, a single‐energy scan of the PLP was also acquired to serve as the clinical reference CBCT protocol.

**TABLE 2:** Cumulative mAs for 80 and 140 kVp at varying framerates across eight DE‐CBCT protocols and PLP (Run 1).

Run	1	2	3	4	5	6	7	8	9
80 kVp mAs	–	4046	4092	3250	3253	2433	2442	2440	2430
140 kVp mAs	1688	833	825	653	660	504	495	483	488
Framerate (s^−1^)	15	15	11	15	11	15	11	7	3

The projections images of DE‐CBCT were first decomposed into projection images of two basis materials of aluminum (Al) and PMMA using the open‐source Tomographic Iterative GPU‐based REconstruction (TIGRE) toolbox.^37^, ^38^ To generate VMIs, the basis material thickness projections were reconstructed using the Feldkamp‐Davis‐Kress (FDK) algorithm to produce basis material images with voxel values corresponding to the relative fraction of each basis material in the CBCT images. The basis material images were then summed and weighted by the attenuation coefficients for Al and PMMA at various VM energies between 40 and 150 keV in 1 keV increment to produce a range of VMIs. Subsequently, post‐processing was applied for Hounsfield unit (HU) mapping and image denoising. A detailed description of VMI generation via material decomposition of DE‐CBCT into equivalent thickness projections of two basis materials can be found in Keeler et al.^39^, ^40^

### 2.3 Image quality assessment

Image quality of VMI was evaluated using three quantitative metrics: (1) relative contrast‐to‐noise ratio (rCNR), (2) HU accuracy, and (3) HU uniformity.

**FIGURE 2:**
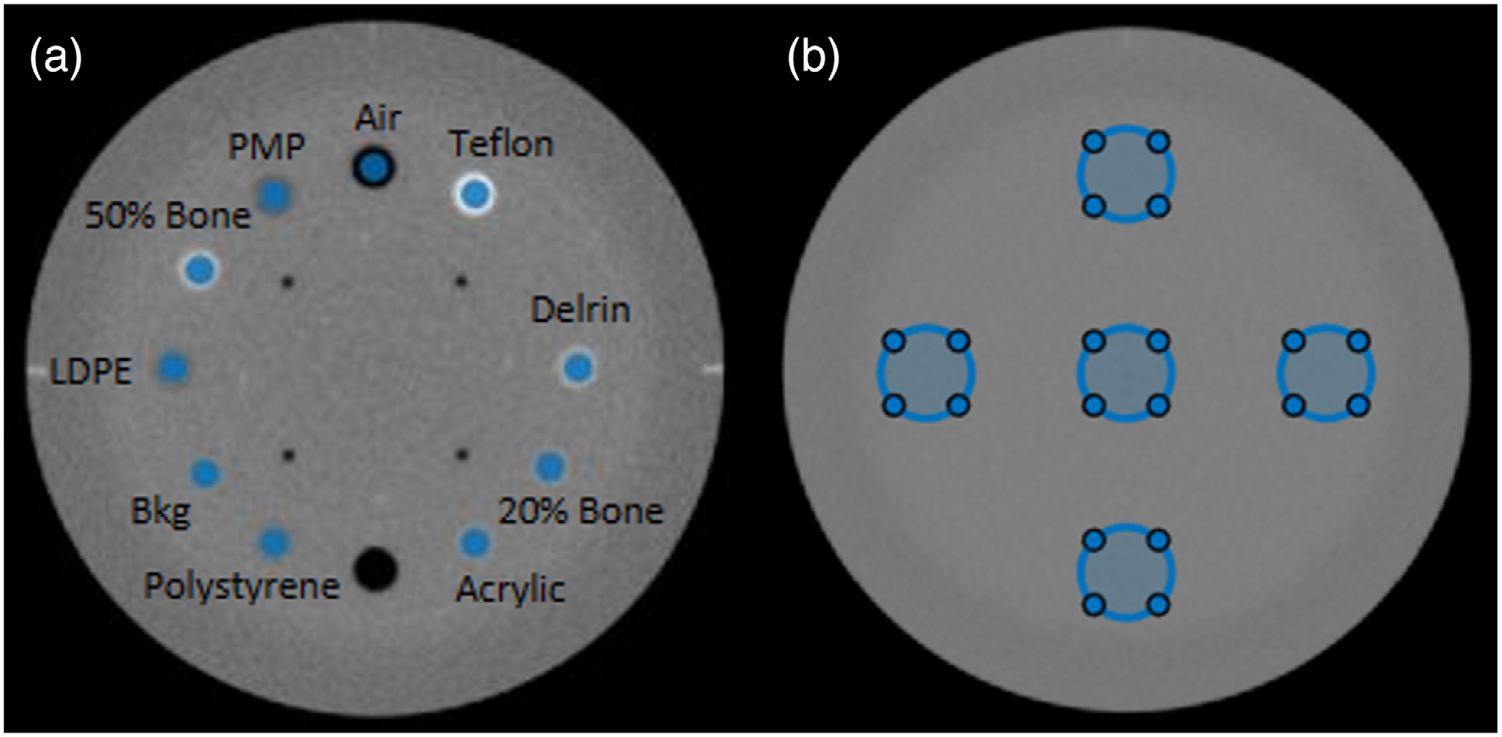
Axial views of Catphan 604 modules with defined regions of interest (ROIs): (a) material inserts used for rCNR and HU accuracy; (b) HU uniformity evaluation.

CNR was calculated for each material insert using the following equation:

(1) $$CNR_{i}=\frac{\left|HU_{i}-HU_{bkg}\right|}{\sqrt{\frac{1}{2}\left(\sigma_{i}^{2}+\sigma_{bkg}^{2}\right)}}$$

where the *i* refers to the material insert, *bkg* refers to the background material of the phantom, HU is the average HU over the regions of interest (ROI) as shown in Figure 2a, and σ is the standard deviation of HU values over the ROI.

The relative CNR (rCNR) for each insert was defined as the ratio of CNR for that insert on the VMI to the corresponding CNR for that insert in the PLP. The mean rCNR for a VMI was computed across all material inserts.

HU accuracy was assessed by comparing the measured HU within the ROIs (Figure 2a) to the corresponding theoretical HU for each material insert. To quantify the HU accuracy, the mean HU differences across all material inserts were calculated.

To assess HU uniformity, five circular ROIs were drawn in the uniformity module of the phantom: four peripheral ROIs located at the top, bottom, left, and right sides of the phantom and one central ROI as shown Figure 2b. The HU uniformity was quantified as the difference between the HU at the center ROI and the mean HU of the four peripheral ROIs.

### 2.4 Dual‐energy image synthesizer for CBCT GUI

Image quality assessment as described above was conducted using an in‐house developed analysis platform, DISC (Dual‐energy Image Synthesizer for CBCT).^40^ DISC is an open‐source, Matlab‐based user interface designed for rapid reconstruction of VMIs and identification of optimal energies from a given set of basis material projections. The platform also displays image quality metrics and statistical data of reconstructed VMI at each energy. The reconstructed VMIs are generated in 1 keV increments across a range of monoenergies with automatically calculated CNR and uniformity on pre‐defined or user‐defined ROIs. These advanced features are possible because DISC employs an efficient computational method which reconstructs basis material images with the voxels representing relative weights of each basis material. VMIs are then rapidly synthesized through a linear summation of voxel‐wise attenuation coefficient weights. In contrast to the TIGRE method, where each VMI is individually reconstructed from weighted basis material projections, this approach substantially expedites the VMI reconstruction, enabling fast optimization of monoenergy selection by maximizing CNR. The VMI generation time was two orders of magnitude faster with our method when generating 130 VMIs between 20 and 150 keV (36.5 s for DISC vs. 2276.1 s for TIGRE). Additionally technical details on DISC framework can be found in Keeler et al.^40^ In this study, DISC was used to identify the CNR‐optimal energy level for each DE‐CBCT acquisition. The image quality metrics of VMIs were evaluated across multiple DE‐CBCT protocols.

## 3 RESULTS

### 3.1 Dose correlations and estimation

**FIGURE 3:**
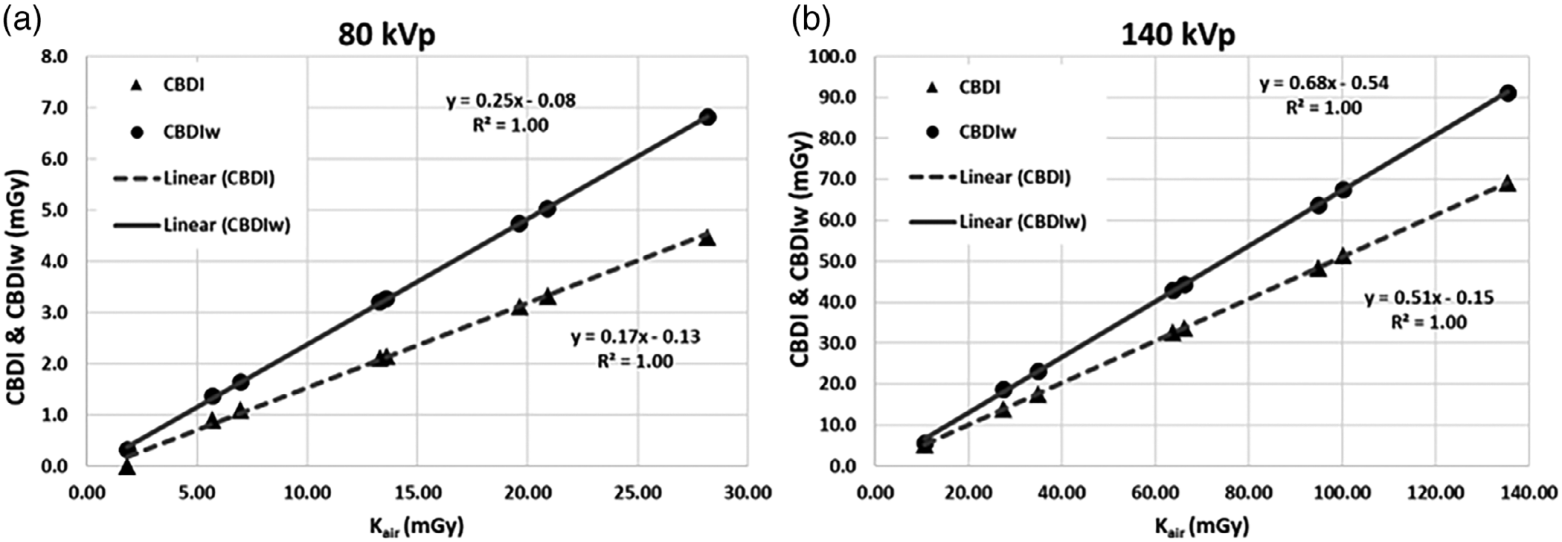
Linear regression functions of measured K_air_ versus CBDI and CBDIw with linear regression functions for (a) 80 kVp and (b) 140 kVp.

**FIGURE 4:**
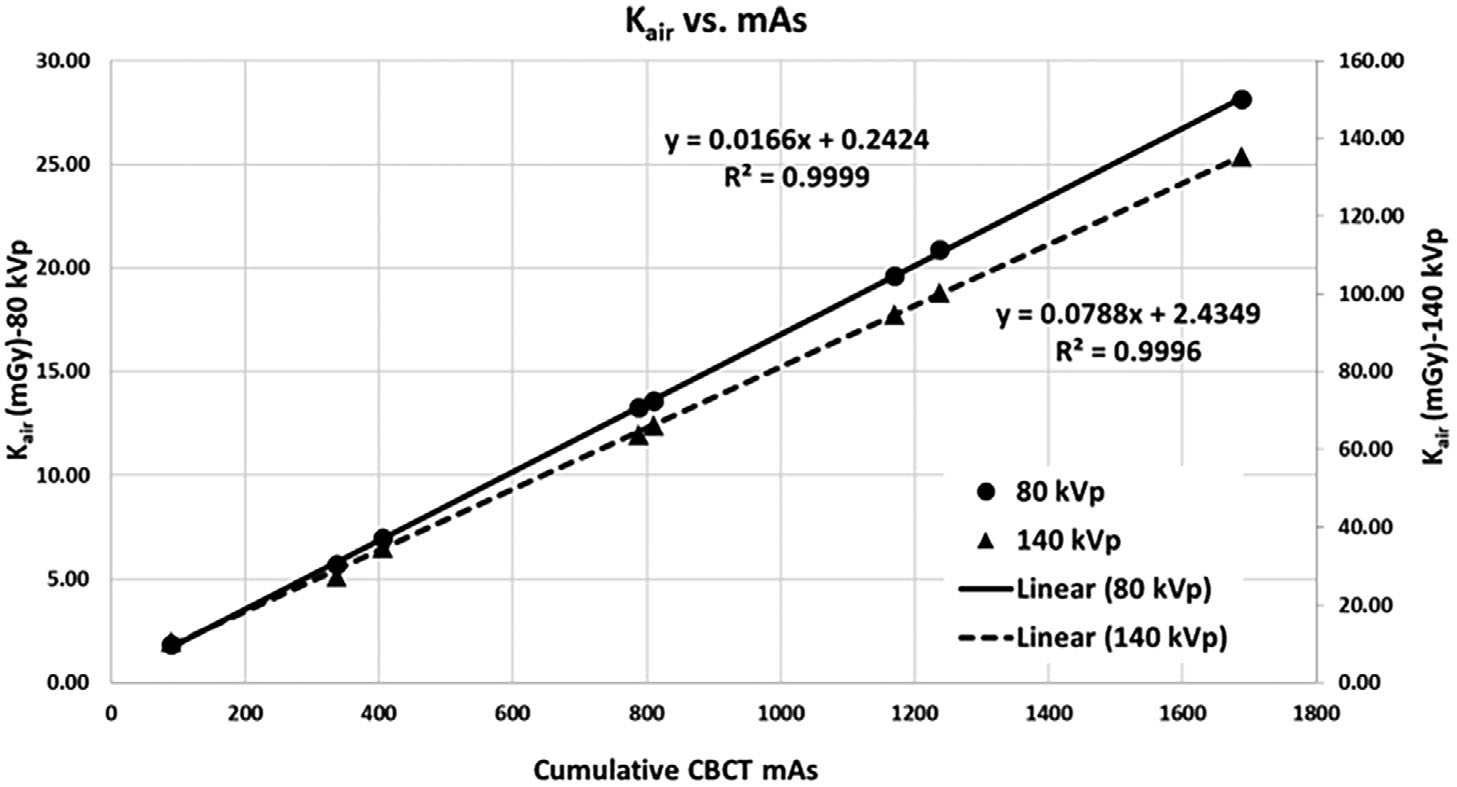
Linear regression functions and measured data of K_air_ versus cumulative CBCT mAs for 80 kVp (solid line & circles). K_air_‐axis on the left side and 140 kVp (dashed line & triangles) K_air_‐axis on the right side.

In Figure 3, the plots of CBDI/CBDIw versus K_air_ for each energy level are presented, and Figure 4 displays K_air_ as a function of cumulative CBCT mAs for both energy levels. Linear regression functions are superimposed in both figures for the respective parameters. Note that the 140 kVp measurements were acquired using a 32‐cm diameter pelvis phantom, whereas the 80 kVp measurements were obtained using a 16‐cm diameter head phantom. To be consistent between the two datasets of imaging dose, a conversion factor of 2.34 (as referenced in [41, p. 213]) was applied to the CBDI and CBDIw values from the 140 kVp data.

Strong positive linear correlations are demonstrated by the regression functions between CBDI/CBDIw and K_air_ at both 80 and 140 kVp, highlighting their interchangeability as quantitative metrics for imaging dose estimation. Additionally, as shown in Figure 4, K_air_ exhibits a strong linear correlation with CBCT mAs at both energy levels. The resulting linear regression functions provide reliable predictions for estimating imaging doses across various DE‐CBCT protocols.

### 3.2 Validation of estimated K_air_‐based imaging doses

Table 3 presents the estimated and measured K_air_ (mGy), along with their percentage difference for both energy levels. Note that DE‐CBCT protocols in Table 3 are the same as those in Table 1 except for Protocol 4 which uses the lowest mAs setting. The K_air_ estimations were derived by substituting the cumulative CBCT mAs values into the linear regression equations shown in Figure 4 for each energy level. Of note, the average difference between estimated and measured K_air_ across seven independent DE‐CBCT protocols was 0.7 ± 2.1% for 80 kVp and 1.3 ± 1.9% for 140 kVp. The largest difference was 5.8% at 338 mAs for 140 kVp. This larger discrepancy at lower cumulative mAs settings is likely due to the limitations of the linear regression models when extrapolated to extremely low mAs. Therefore, caution is advised when applying the estimate method under such scenarios.

**TABLE 3:** Estimated and measured K_air_ of CBCT protocols, showing agreement within 5.8% for both 80 and 140 kVp. *Protocol 4 in Table 1 was excluded because the lowest mAs setting is not typically used in clinical practice.

			80 kVp			140 kVp		
Protocol*	Framerate (s^−1^)	mAs	Estimate (mGy)	Measurement (mGy)	Difference (%)	Estimate (mGy)	Measurement (mGy)	Difference (%)
1	3	338	5.8	5.7	**2.5**	29.0	27.4	**5.8**
2	7	788	13.3	13.3	**0.2**	64.5	63.7	**1.2**
3	11	1238	20.8	20.9	**0.6**	99.9	100.2	**0.2**
5	15	405	7.0	7.0	**0.2**	34.3	34.9	**1.5**
6	15	810	13.7	13.6	**0.6**	66.3	66.2	**0.1**
7	15	1170	19.7	19.6	**0.2**	94.6	94.8	**0.2**
8	15	1688	28.3	28.2	**0.3**	135.4	135.2	**0.1**

### 3.3 Evaluation of VMI image quality

The optimal energy level for VMI, based on rCNR enhancement across eight DE‐CBCT protocols, ranged from 59 to 62 KeV.^40^ Thus, VMIs at 60 keV which was either optimal or near‐optimal for all protocols were generated for quantifying image quality metrics.

Table 4 summarizes the mean rCNR, mean HU accuracy and HU uniformity of VMIs for the PLP and the eight DE‐CBCT. Our analysis results showed that VMIs with the same K_air_ values as the single PLP exhibited substantially improved rCNR, HU accuracy, and HU uniformity, reflecting enhanced image quality as shown Runs 2–3 in Table 4. Moreover, VMIs reconstructed from DE‐CBCT protocols with 60% and 80% K_air_ values displayed enhanced CNR (Runs 4–8 in Table 4) with exception of 3/s framerate. As indicated in Table 4, it was evident that the beam hardening effect was substantially reduced by an order of magnitude for all VMIs compared to clinical CBCT. This reduction is a benefit of VMIs over the PLP, as demonstrated by our results.

**TABLE 4:** Absolute and relative K_air_, mean rCNR, mean HU accuracy and HU uniformity of VMIs at 60 keV from the eight DE‐CBCT protocols and clinical PLP (Run 1). The protocol order is consistent with Table 2.

Run	80kVp mAs	140kVp mAs	Framerate (s^−1^)	K_air_ (mGy)	K_air_ (%)	Mean rCNR (mean ± s.d.)	HU Accuracy (mean ± s.d.)	HU uniformity
1	—	1688**	15	134	100	1.00 (–)​	1.3 ± 18.2	28.6
2	4046	833	15	134	100	1.7 ± 0.3​	0.9 ± 8.9	4.8
3	4092	825	11	134	100	1.2 ± 0.1​	−2.0 ± 9.5	3.2
4	3250	653	15	107	80	1.4 ± 0.2	3.3 ± 8.7	1.5
5	3253	660	11	108	80	1.1 ± 0.1​	0.4 ± 10.6	3.7
6	2433	504	15	82	61	1.3 ± 0.2​	5.4 ± 10.3	4.6
7	2442	495	11	82	61	1.1 ± 0.1​	2.4 ± 8.8	3.2
8	2440	483	7	81	60	1.0 ± 0.2​	1.0 ± 11.2	6.0
9	2430	488	3	81	60	0.5 ± 0.1​	−9.9 ± 9.4	3.7

**FIGURE 5:**
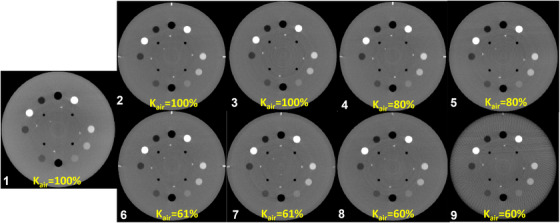
Reference image from the clinical PLP (Run 1) and VMI images at 60 keV from Runs 2 to 9 are shown. The corresponding protocol numbers are listed in Table 2. Window/level are 800/100 HU for all the images. Estimated relative K_air_, as a percentage of the reference image K_air_, is annotated for each VMI. Runs 1–3 correspond to K_air_ = 100%, Runs 4–5 to K_air_ = 80%, and Runs 6–9 to K_air_ = 60%.

**FIGURE 6:**
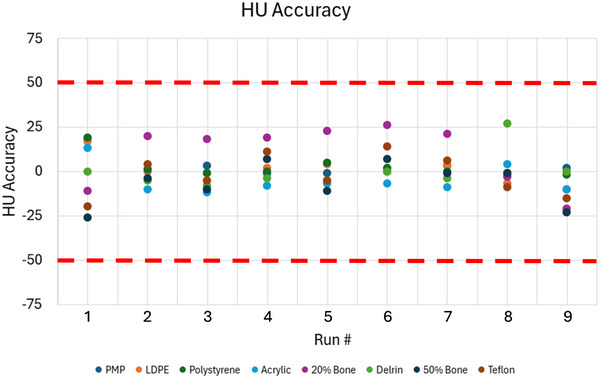
HU accuracy plots across all the material inserts in VMIs from the clinical PLP (Run 1) and eight DE‐CBCT protocols (Runs 2–9). Red lines show manufacturer specification for HU uncertainty, ±50 HU.

The VMIs at different image dose levels between 60% and 100% of the PLP are presented in Figure 5. Note that Figure 5 (Run 9) shows a 3/s framerate VMI exhibiting streak artefacts. Figure 6 shows the HU accuracies of all the material inserts for VMIs from the DE‐CBCT protocols. Qualitatively, all VMIs from the DE‐CBCT protocols exhibited higher HU uniformity at the center compared to the PLP, while maintaining comparable contrasts for the material inserts (Figure 5). HU accuracy was preserved across all reconstructed VMIs demonstrated in Figure 6. The deviations of HU for all inserts across the VMIs remained within 35 HU of theoretical values for all DE‐CBCT protocols.

Our results highlight that VMIs reconstructed from these DE‐CBCT protocols with reduced imaging dose and various framerates resulted in superior image quality compared to the PLP. These findings were consistent across all framerates and imaging dose down to 60% K_air_, except for the 3/s framerate where image quality of VMIs deteriorates substantially with rCNR dropping below 1 and HU accuracy worsening, thus limiting clinical applicability. Also, as shown in Table 4, rCNRs display a dependence on framerate for each K_air_ level, with 15/s framerate being consistently higher than 11/s or 7/s framerates. In contrast, HU accuracy and uniformity does not exhibit a framerate‐dependent trend.

## 4 DISCUSSION

DE‐CBCT offers the potential to improve CBCT imaging in IGRT and possibly in ART by offering enhanced soft tissue contrast and reduced artifacts. However, concerns regarding increased imaging dose from the additional image acquisition for DE‐CBCT necessitate careful dose reduction strategies to mitigate patient exposure. Notably, extensive DE‐CT studies have proposed various dose reduction techniques including post‐processing techniques, split‐filter and dual layer detectors.^42^, ^43^, ^44^, ^45^, ^46^, ^47^, ^48^, ^49^, ^50^ Many commercial scanners now incorporate DE‐CT implementation with imaging dose comparable to single‐energy CT.^44^, ^51^, ^52^, ^53^, ^54^ As DE‐CBCT technology progresses, these advancements in DE‐CT are expected to translate to DE‐CBCT, which is an objective that directly motivates the present study.

This study presents a robust and practical framework for estimating imaging doses and optimizing DE‐CBCT protocols to generate VMIs with enhanced image quality while reducing imaging dose. To address the challenge of optimizing DE‐CBCT protocols based on imaging dose, an imaging dose estimation method using linear regression functions of K_air_ to CBCT mAs was developed. Once the correlations between CBDI/CBDIw, and K_air_ measurements are established, dose estimation becomes straightforward using these functions.^36^ Separate models for low‐ and high‐energy CBCT scans were derived, allowing users to assess and adjust dose allocations between the two energies without requiring direct dosimetric measurements or reliance on literature‐based dose values. Using this approach, DE‐CBCT protocols were identified that achieved dose reductions while maintaining clinical image quality.

Validation of the estimated K_air_ against measured values demonstrated strong agreement, with discrepancies within 2% for the majority of imaging techniques, except one low mAs setting at 140 kVp. The larger variability observed at lower mAs may be attributed to the non‐linearity between the mAs per projection to the dose in very low mAs range, which limits the accuracy of prediction K_air_ depending on whether data points of the low mAs per projection are included in the linear regression model.

Our findings demonstrate the feasibility of generating VMIs with a lower cumulative imaging dose than a standard single CBCT scan while achieving improved rCNR, HU accuracy, and HU uniformity. Given that DE‐CBCT requires two consecutive CBCT scans in our study, this achievement supports the clinical application of DE‐CBCT without raising concerns about additional dose‐related risks of increased chances of secondary cancers. This result is valuable, as balancing image quality and dose has remained a practical and clinically relevant issue. In fact, recent developments have explored the possibility of lowering imaging doses for IGRT while maintaining clinically acceptable image quality for each disease site.^22^, ^23^ To the best of our knowledge, this study is the first to demonstrate that reducing the imaging dose to 60% of a single CBCT scan while simultaneously improving image quality in DE‐CBCT applications. Notably, even at 40% of a single CBCT scan, VMIs showed improved rCNR and HU uniformity with deteriorated HU accuracy (data not shown).

The present study demonstrates that image dose reduction in DE‐CBCT can be effectively achieved within a near‐clinical setting without requiring specialized hardware or software modifications. Sequential DE‐CBCT scans were acquired in treatment mode, consistent with methodologies used in several investigations.^55^, ^56^ By optimizing DE‐CBCT protocols within these settings, we have demonstrated the feasibility of dose‐efficient DE‐CBCT for IGRT applications. These findings support the potential integration of DE‐CBCT into routine clinical workflows where minimizing imaging dose while maintaining image quality is a critical consideration.

Emerging technologies in rapid CBCT acquisition may further enhance the clinical utility of DE‐CBCT. Rapid CBCT acquisition has been commercially available for O‐ring gantry treatment machine and has recently introduced for C‐arm linac (HyperSight, Varian Medical System). HyperSight enables substantially faster image acquisition while maintaining imaging doses that are equivalent or lower than that of CBCT.^57^, ^58^, ^59^, ^60^, ^61^ In the future, our sequential DE‐CBCT method may be readily adaptable to HyperSight, potentially offering reduced imaging times and dose, while mitigating motion artifacts without requiring additional system modifications. In parallel, recent advancements in DE‐CBCT utilizing dual‐layer detectors, fast kV‐switching and split‐filter beam techniques have demonstrated the potential to reduce patient imaging dose while enabling the generation of VMIs from DE‐CBCT.^17^, ^18^, ^20^, ^21^, ^62^, ^63^, ^64^ Although these studies are currently in the research phase, as technical refinement and image‐processing techniques continue to advance, these approaches are expected for clinical implementation. Additionally, AI‐generated DE images from single‐energy images for treatment imaging has been investigated with promising preliminary results, offering a potentially viable alternative of DE‐CBCT acquisitions without any modifications in clinical setting.^65^

However, our approach has several limitations. It is primarily empirical and clinically driven, lacking a comprehensive theoretical framework for the correlations between imaging dose parameters. Moreover, these correlations may need to be re‐established if hardware configurations such as kV source and detector are modified or if new imaging systems are introduced. As highlighted by Li et al.,^29^ our method provides relative dose estimations rather than absolute organ‐specific doses, which limits its applicability in certain contexts. In addition, this study utilized the Catphan phantom for image quality evaluation of VMIs and a 16‐cm diameter head phantom for imaging dose measurements. Consequently, the results may not be directly generalizable to larger pelvis areas. Further validation and potential re‐optimization are warranted for a broader range of parameters, including different scan trajectories (full and half), phantom sizes (head and pelvis) or using patient data to ensure the applicability of the proposed approach.

Also, the observed effect of framerate on enhancement of rCNR in VMIs requires further investigation to understand the underlying mechanism. The image quality of low‐framerate VMI could potentially be recovered using advanced reconstruction techniques such as iterative reconstruction algorithms^66^, ^67^, ^68^ or AI‐based denoising.^69^, ^70^ These advanced methods could facilitate further improvements in image fidelity at reduced doses. Finally, since the sequential DE‐CBCT acquisition in clinical mode takes approximately two minutes, anatomical deformations between low‐ and high‐energy CBCT acquisitions may occur, potentially leading to motion artifacts. To address this limitation technologies such as HyperSight (with substantially reduced acquisition times), dual‐layer detectors and split‐filter approach with simultaneous acquisitions should be explored in the future.

Future work will focus on further reducing imaging doses by lowering integrating advanced reconstruction algorithms to enhance image quality at minimal doses. Additionally, clinical validation of low‐dose DE‐CBCT protocols in patient populations will provide critical insights into their practical applications and benefits.

## 5 CONCLUSION

This study presents a practical, K_air_‐based approach for optimizing DE‐CBCT protocols in IGRT. The method achieves successful image dose‐estimation while leveraging detectors readily available in radiation oncology. Our approach enables robust dose estimation and frequent dose measurement within the radiation oncology department. The application to optimization of DE‐CBCT protocols by balancing enhanced image quality and reduced imaging dose demonstrates its feasibility for clinical translation. It is possible to develop clinical DE‐CBCT protocols with substantially reduced dose by 40% while maintaining image quality comparable to current clinical images, promoting safe and effective imaging practices across clinical settings. Future efforts will focus on refining low‐dose protocols for clinical adoption.

## AUTHOR CONTRIBUTIONS

**Hyejoo Kang**: Initiated and Supervised The Project; Led The Study Design, Data Collection, Data Analysis, And Manuscript Writing. **Andrew Keeler**: Contributed To Data Collection And Analysis; Participated In Manuscript Writing, Review, and Editing. **Matthew Georgesen**: Data Collection And Analysis; Participated In Reviewing and Editing The Manuscript. **Jason Luce**: Analyzing Data, and Reviewing and Editing The Manuscript. **Ha Nguyen**: Analyzing Data, and Reviewing and Editing the Manuscript. **Mathias Lehmann**: Interpreting the Data Analysis and Results, and Reviewing and Editing the Manuscript. **Sebastien Gros**: Designing the Study, Collecting and Analyzing Data, and Reviewing and Editing the Manuscript. **John C. Roeske**: Supervised the Study, Analyzing Data, Interpreting the Results, and Reviewing and Editing the Manuscript.

## CONFLICT OF INTEREST STATEMENT

Mathias Lehmann is an employee of Varian Medical Systems.

## Data Availability

The imaging data of Catphan 604 that support the findings of this study are available from the corresponding author upon reasonable request.
